# Deposition of FeOOH Layer on Ultrathin Hematite Nanoflakes to Promote Photoelectrochemical Water Splitting

**DOI:** 10.3390/mi15030387

**Published:** 2024-03-13

**Authors:** Wenyao Zhang, Ya Zhang, Xiao Miao, Ling Zhao, Changqing Zhu

**Affiliations:** Shandong Provincial Key Laboratory of Optical Communication Science and Technology, School of Physics Science and Information Technology, Liaocheng University, Liaocheng 252059, China

**Keywords:** hematite photoanode, cocatalyst, photoelectrochemical water splitting

## Abstract

Hematite is one of the most promising photoanode materials for the study of photoelectrochemical (PEC) water splitting because of its ideal bandgap with sufficient visible light absorption and stability in alkaline electrolytes. However, owing to the intrinsically high electron-hole recombination, the PEC performance of hematite is still far below that expected. The efficient charge separation can be achieved via growth of FeOOH on hematite photoanode. In this study, hematite nanostructures were successfully grown on the surface of iron foil by the simple immersion deposition method and thermal oxidation treatment. Furthermore, cocatalyst FeOOH was successfully added to the hematite nanostructure surface to improve charge separation and charge transfer, and thus promote the photoelectrochemical water splitting. By utilizing the FeOOH overlayer as a cocatalyst, the photocurrent density of hematite exhibited a substantial 86% increase under 1.5 V_RHE_, while the onset potential showed an apparent shift towards the cathodic direction. This can be ascribed to the high reaction area for the nanostructured morphology and high electrocatalytic activity of FeOOH that enhanced the amount of photogenerated holes and accelerated the kinetics of water splitting.

## 1. Introduction

Photoelectrochemical (PEC) water splitting is a promising method of hydrogen energy production that utilizes the renewable resource of solar energy to split water into hydrogen and oxygen to achieve zero carbon emissions [1,2,3,4,5,6]. Among many photoanodes of metal oxides, hematite (α-Fe_2_O_3_) stands out among various photoanodes due to its low cost, high chemical stability in alkaline electrolytes, high earth abundance, and appropriate bandgap (~2.2 eV) [7,8,9,10]. For hematite, in theory, its solar-to-hydrogen energy conversion efficiencies can reach 16.8% [11,12,13]; however, it is far below this theoretical value in practice. This is mainly due to the low absorption coefficient, short excited-state lifetime (3–10 ps), slow kinetics for the oxygen evolution process, short carrier diffusion length (2–4 nm), and the poor conductivity of hematite (about 10^−2^ cm^2^ V^−1^s^−1^ at 20 °C) [14,15,16,17].

Therefore, numerous researchers have come up with various approaches to solve those problematic flaws above, such as control morphology [18], ion doping [11,14], construction of heterojunction/homojunction [6,15], and deposition of cocatalysts [19,20]. Specifically, the use of nanostructures can shorten the transport distance of the holes, so people have prepared hematite nanostructures with different micromorphology, such as nanowire, nanorods, nanotubes, nanocubes, and nanosheets [20,21,22,23,24]. In order to effectively reduce the surface recombination caused by the surface state, using a cocatalyst is an important means of reducing overpotential, accelerating reaction kinetics, inhibiting corrosion, and accelerating surface charge separation [25,26,27]. Various cocatalyst materials have been studied and deposited to the surface of hematite, including IrO2 [28], cobalt phosphate (Co-Pi) [29], NiFeOx [30], and nickel(II) hydroxide [31]. FeOOH came to the attention of researchers because of its low cost of synthesis and good compatibility with hematite [25,32]. For example, Ye et al. reported that hematite photoanodes decorated with nanostructured FeOOH displayed four times enhancement of photocurrent density in the PEC water oxidation [33]. Bi and co-workers reported the decoration of FeOOH and Au cocatalysts on hematite nanoflake photoanodes showed a photocurrent density of 6.5 mA cm^−2^ at 1.6 V_RHE_ under AM 1.5 G simulated sunlight [25]. Recently, researchers combined bulk and surface engineering to change the photocurrent density of hematite photoanodes. Mai et al. introduced a Ti dopant into Fe_2_O_3_ crystal structure to enhance charge separation efficiency and bulk kinetics, and deposited FeOOH on the Ti-doped Fe_2_O_3_ to promote the charge transfer efficiency and surface kinetics [34]. FeOOH can facilitate the kinetics of water oxidation as an oxygen evolution cocatalyst, which has widely been utilized in photoanodes [35,36,37,38,39].

In this study, in order to shorten the hole transport distance, we successfully grew the nanoflake structure on the surface of the iron foil. In order to solve the problem that a large number of photogenerated holes are accumulated on the surface of photoanode materials due to slow oxygen generation kinetics, we successfully loaded the cocatalyst FeOOH on the surface of the photoanode to improve the transfer efficiency of the photogenerated holes. The loaded FeOOH layer provides a way of avoiding the accumulation of holes on the surface of photoanode materials, so that the holes can effectively participate in the water oxidation reaction and improve the oxygen evolution reaction. Therefore, the micromorphology and surface modification of the sample have certain influence on the PEC performance of hematite, which provides an inspiration for designing efficient photoelectrode devices.

## 2. Materials and Methods

### 2.1. Preparation of α-Fe_2_O_3_ and α-Fe_2_O_3_/FeOOH Nanosheets

We prepared hematite on iron foil (99.99%, Alfa Aesar, Haverhill, MA, USA), which was degreased by sonicating in acetone and ethanol for 10 min, and then dried in an air stream. Then, the iron foil was directly placed in the muffle furnace (KSL-1100X, HFKejing furnace, Hefei, China) in air at 450 °C, and maintained at the desired temperature for 1.5 h with a heating rate of 10 °C min^−1^. As for the preparation of α-Fe_2_O_3_/FeOOH samples, primarily, the iron foils were immersed in 5 mM FeCl_3_·6H_2_O (99%, Aladdin, Shanghai, China) aqueous solution for 10 min, 20 min, 30 min, 50 min, and 60 min, following by rinsing with ultrapure water and drying in an air stream. In the control group, the iron foils were soaked in 10 mM FeCl_3_·6H_2_O (99%, Aladdin) aqueous solution for 10 min, 20 min, 30 min, 50 min, and 60 min, following by rinsing with ultrapure water and drying in an air stream. After that, the samples were thermally annealed in a muffle furnace (KSL-1100X, HFKejing furnace) in air at 450 °C, maintained at the desired temperature for 1.5 h, and the heating rate was 10 °C min^−1^. The samples were taken out of the furnace when the temperature in the furnace decreased to room temperature. In the following, α-Fe_2_O_3_/FeOOH refers to the samples obtained after the iron foil was immersed in 5 mM FeCl_3_·6H_2_O (99%, Aladdin) aqueous solution for 30 min for annealing.

### 2.2. Material Characterization

The pristine α-Fe_2_O_3_ and α-Fe_2_O_3_/FeOOH samples were examined for surface morphology using a thermal field emission scanning electron microscope (MERLIN compact, Oberkochen, Germany). Using a 532 nm laser as the excitation source, a Raman spectrometer (iHR 550 HORIBA, Kyoto, Japan) was used to record the Raman spectra of hematite that was soaked and unsoaked for varying durations. X-ray photoelectron spectroscopy (XPS, Escalab Xi+, Waltham, MA, USA) was utilized to illustrate the FeOOH layer on hematite and determine the surface composition of the pristine α-Fe_2_O_3_ and α-Fe_2_O_3_/FeOOH samples.

### 2.3. Photoelectrochemical Performance Measurements

The PEC performance experiments used a three-electrode configuration. A saturated calomel electrode (SCE) was employed as the reference electrode, platinum mesh was used for the counter electrode, and the prepared α-Fe_2_O_3_ and α-Fe_2_O_3_/FeOOH photoanodes served as the working electrodes in all PEC examinations. An electrolyte solution of 1M NaOH (pH 13.6) was utilized. Stirring was used throughout the entire PEC measuring process. Without the use of a light filter, a 500 W xenon lamp with a light intensity of 225 mW cm^−2^ was used as the light source.

A CHI660E potentiostat (CH Instruments, Austin, TX, USA) was implemented for determining the photocurrent density using linear sweep voltammetry (LSV) at a scan rate of 10 mV s^−1^. The equation *E*_RHE_ = *E*_SCE_ + 0.059 pH + *E*^0^_SCE_, where E_SCE_ is the experimentally observed potential against the reference electrode and *E*^0^_SCE_ = 0.245 V at ambient temperature, was used to convert the measured potentials versus SCE to the reversible hydrogen electrode (RHE) scale. In 1 M NaOH electrolyte, Mott–Schottky experiments were carried out using the same photoelectrochemical apparatus in a dark environment. The scan rate was 50 mV s^−1^ and the measurement frequency was 1000 Hz. Electrochemical impedance spectroscopy (EIS) measurements were carried out at 10,000 to 0.1 Hz using 0.5 V versus SCE in the dark.

## 3. Results and Discussion

**Figure 1:**
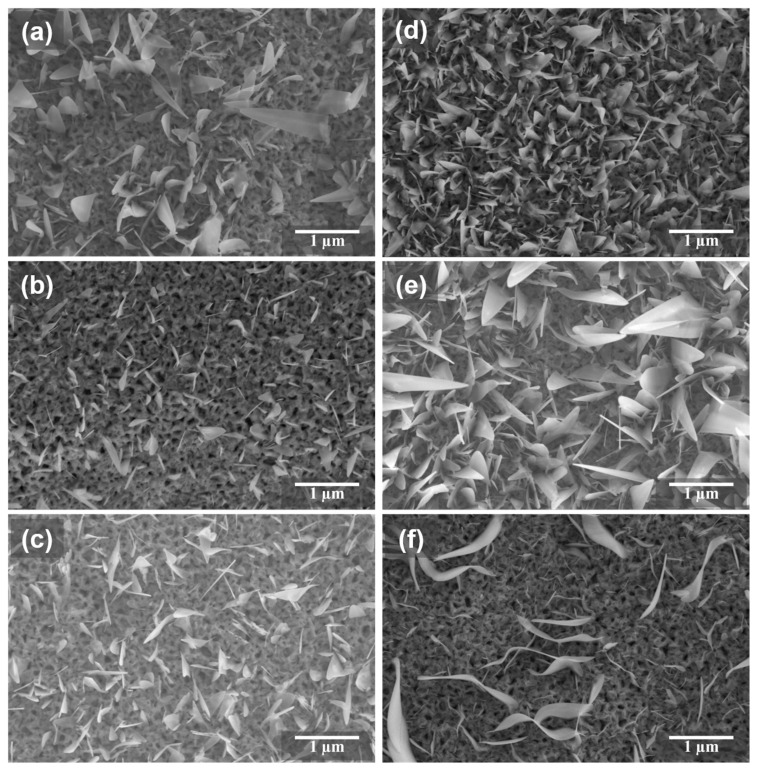
The morphology of iron foil samples sintered in air for 1.5 h at the same temperature with soaking and without soaking, respectively. (**a**) Iron foil annealing in air at 450 °C for 1.5 h. (**b**–**f**) iron foil soaked in 5 mM FeCl_3_ solution for 10 min, 20 min, 30 min, 50 min, and 60 min, respectively; then, annealed in air at 450 °C for 1.5 h.

The morphology of α-Fe_2_O_3_ nanoflakes and hematite modified with the FeOOH cocatalyst is shown in Figure 1. It can be clearly seen that α-Fe_2_O_3_ nanoflakes appear, but it can also be seen that the distribution of the nanoflakes is not uniform and is relatively sparse, as shown in Figure 1a. In combined immersion deposition and thermal oxidation treatment in air, the morphology of samples makes a difference. Figure 1b shows the SEM image of the α-Fe_2_O_3_/FeOOH combined with immersion for 10 min and sintering in the muffle furnace at 450 °C for 1.5 h. One can find that there are less nanoflakes than in the sample of α-Fe_2_O_3_. However, as shown in Figure 1c, the nanoflakes gradually increase under the immersion time of 20 min. Once the immersion time reaches 30 min, the sample shows uniformly distributed nanoflakes, which can be seen in Figure 1d. The length of hematite nanoflakes is about 1–2 μm and the thickness of the flakes tapers down from the base to the tip. Similarly, in Figure 1e, the sample still exhibits dense nanoflakes under the immersion time of 50 min. Nevertheless, from Figure 1f, the nanoflakes decrease drastically when the immersion time reaches 60 min. In consequence, a suitable immersion time plays an important role in forming uniformly distributed nanoflakes before sintering. It can be seen that after the optimized decoration cocatalyst FeOOH modification, the nanoflakes are much denser than the pristine hematite sample. From the surface morphology of hematite, one can find that the uniformly distributed nanoflakes were obtained after proper immersion treatment. Indeed, in a previous study, pre-polishing was necessary to obtain dense nanoflakes for an iron foil substrate [40]. Therefore, the suitable decoration cocatalyst FeOOH on hematite can not only make the nanoflakes dense and uniform on the iron surface, but also change the surface area of the sample for surface reaction and shorten the distance of hole transport.

**Figure 2:**
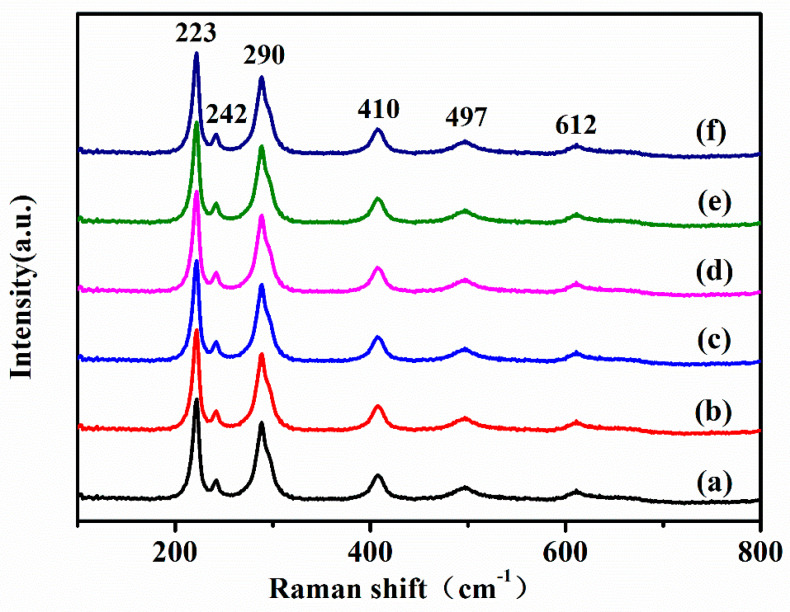
Raman spectra of hematite sintered under various soaking times and at the same thermal oxidation temperature in air. Sample (**a**) was the original control group (annealing at 450 °C for 1.5 h), and samples (**b**–**f**) were soaked for 10 min, 20 min, 30 min, 50 min, and 60 min, respectively.

Figure 2 displays the Raman spectra of hematite samples sintered under different conditions. Sample (a) was the original control group (annealing at 450 °C for 1.5 h), and samples (b)–(f) were soaked for 10 min, 20 min, 30 min, 50 min, and 60 min, respectively. Each sample displays the identical hematite characteristic peak in the range of 200–800 cm^−1^. Hematite belongs to the crystallographic space groups, which include A_1g_ modes (223 cm^−1^ and 497 cm^−1^) and E_1g_ modes (242, 290, 410, and 612 cm^−1^) [41,42].

**Figure 3:**
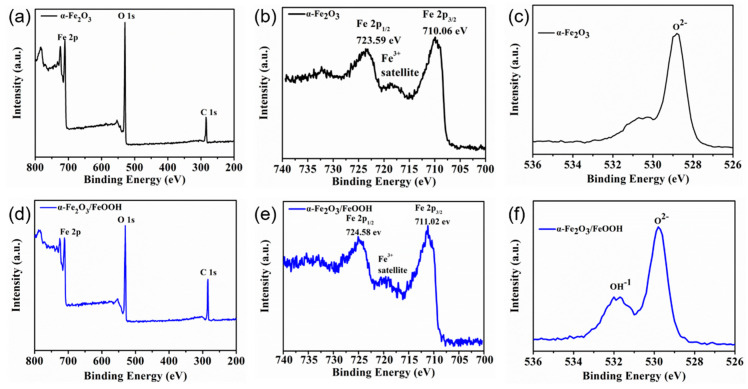
X-ray photoelectron spectroscopy analysis: (**a**) survey spectra of the as-grown hematite; (**b**) Fe 2p peaks of the as-grown hematite; (**c**) O 1s peaks of the as-grown hematite; (**d**–**f**) survey spectra, Fe 2p peaks, and O 1 s peaks of α-Fe_2_O_3_/FeOOH, respectively.

X-ray photoelectron spectroscopy was used to discern whether the pristine hematite and the modified hematite were covered by the FeOOH film. All samples were calibrated with a standard carbon peak of 284.6 eV. Figure 3 presents a summary of the XPS findings for the hematite and α-Fe_2_O_3_/FeOOH samples. The Fe 2p spectra of the as-grown sample (Figure 3b) exhibits a typical hematite spectrum with binding energies of 710.6 eV for Fe 2p3/2 and 723.59 eV for Fe 2p1/2. Additionally, there is a shakeup satellite at 718 eV, which corresponds to the characteristic satellite for phases of α-Fe_2_O_3_ or γ-Fe_2_O_3_. The Raman spectra analysis excluded the potential existence of γ-Fe_2_O_3_, as no vibration mode associated with γ- Fe_2_O_3_ was observed. In Figure 3e, the FeOOH-decorated α- Fe_2_O_3_ sample exhibits signal peaks at 711.02 eV and 724.8 eV, which correspond to Fe 2p_3/2_ and Fe 2p_1/2_, respectively. These results are consistent with the valence state of Fe^3+^ in typical α-Fe_2_O_3_ [43,44]. Figure 3c,f show the O 1 s spectra of as-grown hematite and the FeOOH decoration on α-Fe_2_O_3_ samples. After the addition of the cocatalyst, the peak of O 1 s can be divided into two peaks. The peaks at 529.8 eV and 531.68 eV correspond to the Fe-O peak in Fe_2_O_3_ and the Fe-OH peak in FeOOH, respectively. The signal peak located at 529.8 eV corresponds to the lattice oxygen in Fe_2_O_3_ [32]. The above results are also consistent with those reported in other studies in the literature [45,46].

**Figure 4:**
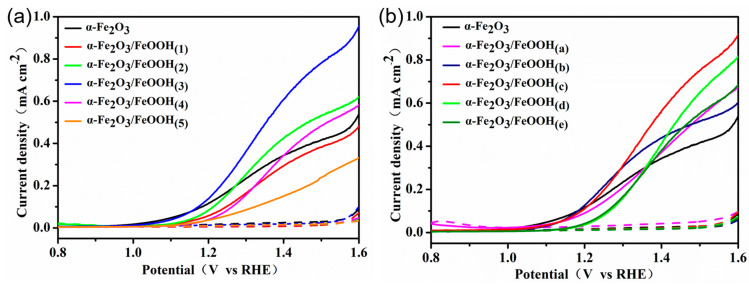
*J*-*V* curves of hematite and α-Fe_2_O_3_/FeOOH_(x)_ samples. (**a**) The α-Fe_2_O_3_/FeOOH_(x)_ samples were immersed in 5 mM FeCl_3_ for different times: α-Fe_2_O_3_/FeOOH_(1)_: 10 min; α-Fe_2_O_3_/FeOOH_(2)_: 20 min; α-Fe_2_O_3_/FeOOH_(3)_: 30 min; α-Fe_2_O_3_/FeOOH_(4)_: 50 min; α-Fe_2_O_3_/FeOOH_(5)_: 60 min. (**b**) The α-Fe_2_O_3_/FeOOH_(x)_ samples were immersed in 10 mM FeCl_3_ for different times: α-Fe_2_O_3_/FeOOH_(a)_: 10 min; α-Fe_2_O_3_/FeOOH_(b)_: 20 min; α-Fe_2_O_3_/FeOOH_(c)_: 30 min; α-Fe_2_O_3_/FeOOH_(d)_: 50 min; α-Fe_2_O_3_/FeOOH_(e)_: 60 min. PEC water splitting experiments were carried out in 1 M NaOH electrolyte under Xenon lamp with illumination irradiance of 225 mW cm^−2^. Solid and dashed lines represent illuminated and dark conditions, respectively.

The α-Fe_2_O_3_/FeOOH nanostructures were prepared by combining the immersion deposition method and thermal oxidation treatment. The surface of the iron sheet was formed as an ultrathin and dense FeOOH film for decoration to enhance the hematite photoanode PEC water oxidation performance. Utilizing a xenon lamp (225 mW cm^−2^) to measure the photocurrent density with bias potential in 1 M NaOH electrolyte, the PEC water oxidation activity of several photoanodes was investigated, as shown in Figure 4.

According to Figure 4a, the as-grown α-Fe_2_O_3_ nanoarrays exhibited a water oxidation initial potential of 0.9 V when the potential was swept from 0.8 V to 1.6 V under illumination, and the current density was 0.15 mA/cm^2^ at 1.23 V. For the α-Fe_2_O_3_/FeOOH_(3)_ photoanode, it is evident that there is an obvious enhanced photoresponse from 1.1 V to 1.6 V, and the photocurrent density is up to 0.2 mA/cm^2^ at 1.23 V, while there is a 1.33 times enhancement of the photocurrent density compared to the as-grown hematite. Moreover, it can be seen that the photocurrent density increases significantly and can reach 0.78 mA/cm^2^ at 1.5V, which is 1.9 times that of the as-grown hematite (0.42 mA/cm^2^). By adjusting the immersion period, the quantities of FeOOH on the hematite nanoflakes could be customized. Figure 4a also shows the solar water splitting performance of α-Fe_2_O_3_/FeOOH photoanodes. It is evident that the photocurrent density increases with lowering the amount of FeOOH (20 min), particularly for bias potentials above 1.3 V. However, the data indicate a decline in photocurrent density between 0.9 and 1.25 V when compared to the as-grown hematite. Compared to the as-prepared hematite, a further immersion time increase of up to 50 min improves the photoresponse; however, it is still less than the optimal time of 30 min. Furthermore, it is evident that the photocurrent density decreases with soaking times of 10 and 60 min compared to pristine hematite.

**Figure 5:**
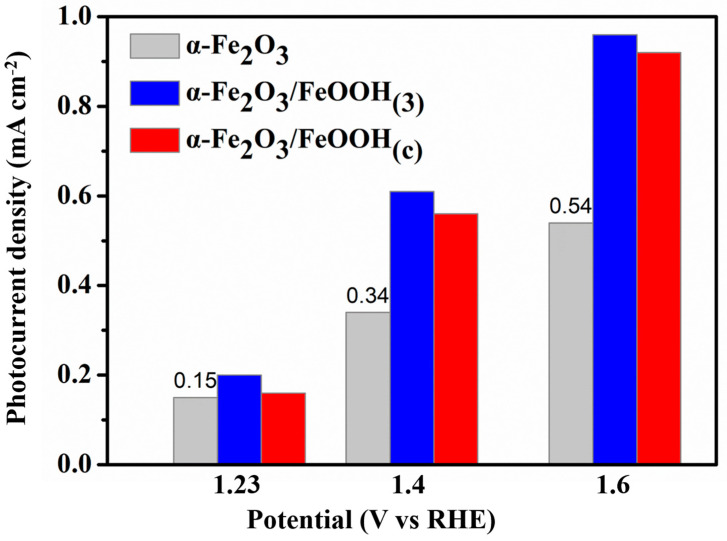
Comparison of photocurrent densities under different applied bias potential for α-Fe_2_O_3_, α-Fe_2_O_3_/FeOOH_(3)_, and α-Fe_2_O_3_/FeOOH_(c)_, respectively.

In order to explore the influence of FeCl_3_, 10 mM FeCl_3_ was selected for comparison. After an equal immersion time in 10 mM FeCl_3_ and then sintering in the same conditions, the PEC water splitting performance is exhibited in Figure 4b. One can find that α-Fe_2_O_3_/FeOOH_(c)_ shows the optimal photocurrent density during the measured applied bias potential. Its photocurrent density is at 1.23 V up to 0.56 mA cm^−2^, and a maximum photocurrent density of 0.92 mA cm^−2^ is obtained at 1.6 V. In order to provide a more visual comparison of the influence of immersion concentration, Figure 5 summarizes the photocurrent density of α-Fe_2_O_3_, α-Fe_2_O_3_/FeOOH_(3)_, and α-Fe_2_O_3_/FeOOH_(c)_ under the potential of 1.23 V, 1.4 V, and 1.6 V, respectively. Obviously, the α-Fe_2_O_3_/FeOOH_(3)_ exhibits the optimal PEC water splitting performance. Therefore, a suitable FeOOH amount on the hematite photoanodes results in an improvement in the PEC water oxidation.

**Figure 6:**
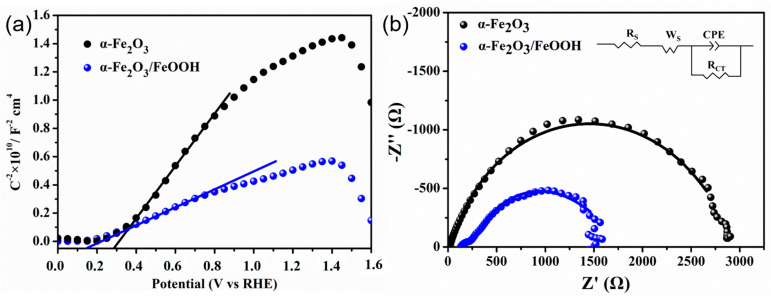
(**a**) Mott-Schottky plots of α-Fe_2_O_3_ and α-Fe_2_O_3_/FeOOH. Mott-Schottky measurements were performed in 1 M NaOH solution at a frequency of 1 kHz under dark environment. (**b**) Electrochemical impedance spectroscopy (EIS) of hematite. EIS measurements were performed in 1 M NaOH at 0.5 V and in the frequency ranging from 10,000 to 1 Hz. Black circles represent α-Fe_2_O_3_ photoanode, and blue circles represent α-Fe_2_O_3_/FeOOH photoanode.

To conduct further electrochemical characterization, Mott–Schottky (M-S) analysis was carried out, as depicted in Figure 6a. The flat band potential and donor density can be ascertained using the M-S measurement. Both the α-Fe_2_O_3_ and α-Fe_2_O_3_/FeOOH photoanodes exhibit positive slopes, indicating their n-type semiconductor characteristic with electrons as the majority carriers. As for the flat band potential, which was derived from the intercept of the x-axis, it can be seen that 0.14 V shifts to the cathodic direction. The FeOOH layer may be responsible for the shift in the flat band potential due to the passivation of surface states. Surface states induce a decrease in potential within the Helmholtz layer; however, this effect can be reduced through surface passivation. The FeOOH overlayer can efficiently passivate the surface of the nanoflakes by creating a wider contact area.

(1) $$\frac{1}{C^{2}}=\left(\frac{2}{e\varepsilon_{0}\varepsilon N_{d}}\right)\left[\left(V-V_{\mathrm{fb}}\right)-\frac{kT}{e}\right]$$

The above Mott–Schottky equation can be used to estimate the carrier density [11,40]. It involves the specific capacitance (*C*), electron charge (*e*), permittivity of vacuum (*ε*_0_), dielectric constant of hematite (*ε* = 80), donor density (*N*_d_), applied voltage (*V*), flat band potential (*V*_fb_), Boltzmann constant (*k*_B_), and absolute temperature (*T*). The donor density is derived from the gradient calculated in the Mott−Schottky plots:

(2) $$N_{d}=\frac{2}{e\varepsilon_{0}\varepsilon }{\left[\frac{d\left(\frac{1}{C^{2}}\right)}{dV}\right]}^{-1}$$

The carrier concentrations of α-Fe_2_O_3_/FeOOH and α-Fe_2_O_3_ were calculated to be 1.48 × 10^19^ cm^−3^ and 4.88 × 10^18^ cm^−3^, respectively. After adding the cocatalyst, the carrier concentration of the photoanode material increased. The α-Fe_2_O_3_/FeOOH structure exhibits a lower flat band potential and a larger carrier density, indicating that the formation of ultrathin FeOOH films could enhance the process of charge separation and transfer.

Electrochemical impedance spectroscopy was used to analyze the process taking place at the interface between the photoelectrodes and the electrolyte. The measurements were conducted under dark conditions. Figure 6b displays Nyquist plots of α-Fe_2_O_3_ and α-Fe_2_O_3_/FeOOH. The inset in the upper right corner represents the equivalent circuit, where R_s_ denotes the series resistance, R_ct_ and CPE represent the charge transfer resistance and the capacitance of the depletion layer, respectively, and W_s_ represents the Warburg impedance. The fitted curves, shown by solid lines, exhibit a high level of agreement with the measured points, which are denoted by symbols. The simulated results show that R_CT_ and CPE of α-Fe_2_O_3_ are 2713 Ω and 1.15 × 10^−4^ F, respectively, and those of α-Fe_2_O_3_/FeOOH are 1027 Ω, and 3.56 × 10^−4^ F. While comparing the hematite nanoflake photoanode with the sample covered in FeOOH film, it was observed that the charge transfer resistance decreased and the capacitance increased. This suggests that the FeOOH layer on the hematite reduced the charge transfer resistance and improved the capacitance at the interface between the photoanode and electrolyte.

**Figure 7:**
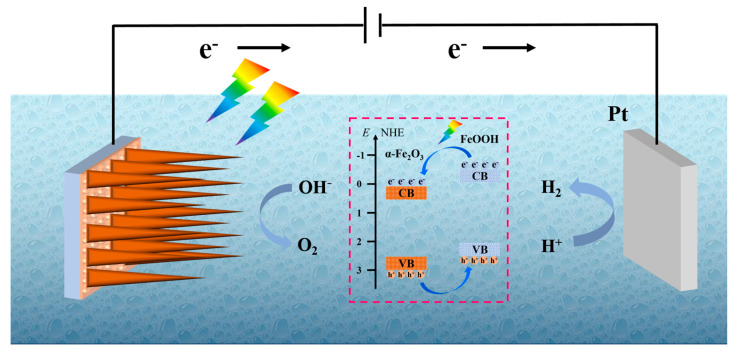
Schematic representation of charge separation and transfer on α-Fe_2_O_3_/FeOOH nanoflakes photoanode.

In this work, we tried to use the combination of immersion pre-treating the iron foil and annealing in an air environment, which exhibits a more efficient PEC water splitting performance for α-Fe_2_O_3_/FeOOH. On the basis of the above results, a possible mechanism for illustrating the photogenerated charge transfer on α-Fe_2_O_3_/FeOOH is displayed in Figure 7. Under light illumination, hematite can be excited to induce photogenerated electrons to leap into the conduction band; meanwhile, photogenerated holes form on the valance band. The photoinduced electrons favorably transfer from the conduction band of hematite to the iron substrate via the nanoflake structure, then to the Pt electrode through an external circuit for hydrogen evolution reaction. The photoinduced holes transfer from the valence band of hematite to the interface of the photoanode/electrolyte to participate in the water oxidation reaction. However, during the photogenerated charge transport, the separated photogenerated charges rapidly recombine with each other during the short hole-diffused length of hematite. As for the α-Fe_2_O_3_/FeOOH photoanode, there is the ultrathin FeOOH layer to decorate the hematite surface. The FeOOH layer can serve as an oxygen evolution cocatalyst. It could make the photogenerated holes on the valence band of hematite transfer to the FeOOH layer [47,48,49], to finish the water oxidation and produce oxygen, which is shown in the inset of Figure 7. On the other hand, the FeOOH facilitates the suppression of the formation of the surface recombination centers. As a result, the PEC water splitting performance of α-Fe_2_O_3_/FeOOH is enhanced.

## 4. Conclusions

In summary, a hematite nanostructure was successfully prepared on iron foil by immersion precipitation and the thermal oxidation treatment method. Different immersion times and concentrations were explored to ensure a suitable FeOOH amount. The results demonstrate that the suitable FeOOH amount decorated on hematite photoanodes could make the nanoflakes dense and uniform on the iron surface and result in an improvement in PEC water oxidation. When the bias voltage is 1.5 V, the photocurrent density of the α-Fe_2_O_3_/FeOOH photoanode is increased by about 86% compared with that of the pristine photoanode. The FeOOH layer on the photoanode surface provides an alternative way to avoid the accumulation of holes on the α-Fe_2_O_3_ photoanode surface, so that the holes can effectively participate in the water oxidation process. The surface modification of FeOOH can effectively promote the oxygen evolution reaction of semiconductor α-Fe_2_O_3_ and improve the charge transfer efficiency. As a result, we identified a simple way to obtain large-area hematite nanoflakes on iron foil and then further to use FeOOH to enhance the photoelectrochemical performance of hematite.

## References

[1] Liu Y., Yu Y.X., Zhang W.D. (2012). Photoelectrochemical properties of Ni-doped Fe_2_O_3_ thin films prepared by electrodeposition. Electrochim. Acta.

[2] Gong M., Li Y., Wang H., Liang Y., Wu J.Z., Zhou J., Wang J., Regier T., Wei F., Dai H. (2013). An advanced Ni-Fe layered double hydroxide electrocatalyst for water oxidation. J. Am. Chem. Soc..

[3] Sivula K., Formal F.L., Grätzel M. (2011). Solar water splitting: Progress using hematite (alpha-Fe_2_O_3_) photoelectrodes. ChemSusChem.

[4] Hou Y., Zuo F., Dagg A., Feng P. (2012). Visible light-driven alpha-Fe_2_O_3_ nanorod/graphene/BiV_1−x_Mo_x_O_4_ core/shell heterojunction array for efficient photoelectrochemical water splitting. Nano Lett..

[5] Mishra M., Chun D. (2015). α-Fe_2_O_3_ as a photocatalytic material: A review. Appl. Catal. A Gen..

[6] Hong Y., Jeon S., Jeong H., Ryu H. (2021). Systematic study of α-Fe_2_O_3_ stacked homojunction photoelectrochemical photoelectrode. Ceram. Int..

[7] Xu X., Pan L., Zhang X., Wang L., Zou J. (2019). Rational design and construction of cocatalysts for semiconductor-based photo-electrochemical oxygen evolution: A comprehensive review. Adv. Sci..

[8] Tilley S.D. (2019). Recent Advances and emerging trends in photo-electrochemical solar energy conversion. Adv. Energy Mater..

[9] Tamirat A.G., Rick J., Dubale A.A., Sub W.N., Hwang B.J. (2016). Using hematite for photoelectrochemical water splitting: A review of current progress and challenges. Nanoscale Horiz..

[10] Xi L., Bassi P.S., Chiam S.Y., Mak W.F., Tran P.D., Barber J., Loo J.S.C., Wong L.H. (2012). Surface treatment of hematite photoanodes with zinc acetate for water oxidation. Nanoscale.

[11] Ling Y., Wang G., Wheeler D.A., Zhang J.Z., Li Y. (2011). Sn-doped hematite nanostructures for photoelectrochemical water splitting. Nano Lett..

[12] Gurudayal, Sabba D., Mulmudi H.K., Wong L., Barber J., Grätzel M., Mathews N. (2015). Perovskite–hematite tandem cells for efficient overall solar driven water splitting. Nano Lett..

[13] Lin Y., Yuan G., Sheehan S., Zhou S., Wang D. (2011). Hematite-based solar water splitting: Challenges and opportunities. Energy Environ. Sci..

[14] Luo Z., Li C., Liu S., Wang T., Gong J. (2017). Gradient doping of phosphorus in Fe_2_O_3_ nanoarray photoanodes for enhanced charge separation. Chem. Sci..

[15] Bai S., Chu H., Xiang H., Luo R., He F., Chen A. (2018). Fabricating of Fe_2_O_3_/BiVO_4_ heterojunction based photoanode modified with NiFe-LDH nanosheets for efficient solar water splitting. Chem. Eng. J..

[16] Kment S., Riboni F., Pausova S., Wang L., Han H., Hubicka Z., Krysa J., Schmuki P., Zboril R. (2017). Photoanodes based on TiO_2_ and alpha-Fe_2_O_3_ for solar water splitting—Superior role of 1D nanoarchitectures and of combined heterostructures. Chem. Soc. Rev..

[17] Forster M., Potter R.J., Ling Y., Yang Y., Klug D.R., Li Y., Cowan A.J. (2015). Oxygen deficient alpha-Fe_2_O_3_ photoelectrodes: A balance between enhanced electrical properties and trap-mediated losses. Chem. Sci..

[18] Kim J.Y., Magesh G., Youn D.H., Jang J.W., Kubota J., Domen K., Lee J.S. (2013). Single-crystalline, wormlike hematite photoanodes for efficient solar water splitting. Sci. Rep..

[19] Qi Y., Zhang J., Kong Y., Zhao Y., Chen S., Li D., Liu W., Chen Y., Xie T., Cui J. (2022). Unraveling of cocatalysts photodeposited selectively on facets of BiVO_4_ to boost solar water splitting. Nat. Commun..

[20] Ye K., Wang Z., Li H., Yuan Y., Huang Y., Mai W. (2018). A novel CoOOH/(Ti, C)-Fe_2_O_3_ nanorod photoanode for photoelectrochemical water splitting. Sci. China Mater..

[21] Tang P., Xie H., Ros C., Han L., Biset-Peiró M., He Y., Kramer W., Rodríguez A.P., Saucedo E., Galán-Mascarós J.R. (2017). Enhanced photoelectrochemical water splitting of hematite multilayer nanowire photoanodes by tuning the surface state via bottom-up interfacial engineering. Energy Environ. Sci..

[22] Schrebler R., Ballesteros L.A., Gomez H., Grez P., Cordova R., Munoz E., Schrebler R., Ramos-Barrado J.R., Dalchiele E.A. (2014). Electrochemically grown self-organized hematite nanotube arrays for photoelectrochemical water splitting. J. Electrochem. Soc..

[23] Zheng C., Zhu Z., Wang S., Hou Y. (2015). NaF-assisted hydrothermal synthesis of Ti-doped hematite nanocubes with enhanced photoelectrochemical activity for water splitting. Appl. Surf. Sci..

[24] Xie X., Li K., Zhang W.D. (2016). Photoelectrochemical properties of Ti-doped hematite nanosheet arrays decorated with CdS nanoparticles. RSC Adv..

[25] Wang L., Hu H., Nguyen N.T., Zhang Y., Schmuki P., Bi Y. (2017). Plasmon-induced hole-depletion layer on hematite nanoflake photoanodes for highly efficient solar water splitting. Nano Energy.

[26] Chen D., Liu Z., Zhang S. (2020). Enhanced PEC performance of hematite photoanode coupled with bimetallic oxyhydroxide NiFeOOH through a simple electroless method. Appl. Catal. B Environ..

[27] Liu W., Liu H., Dang L., Zhang H., Wu X., Yang B., Li Z., Zhang X., Lei L., Jin S. (2017). Amorphous cobalt-iron hydroxide nanosheet electrocatalyst for efficient electrochemical and photo-electrochemical oxygen evolution. Adv. Funct. Mater..

[28] Tilley S.D., Cornuz M., Sivula K., Grätzel M. (2010). Light-Induced Water Splitting with Hematite: Improved Nanostructure and Iridium Oxide Catalysis. Angew. Chem. Int. Ed..

[29] Klahr B., Gimenez S., Fabregat-Santiago F., Bisquert J., Hamann T.W. (2012). Photoelectrochemical and Impedance Spectroscopic Investigation of Water Oxidation with “Co−Pi” Coated Hematite Electrodes. J. Am. Chem. Soc..

[30] Du C., Yang X., Mayer M.T., Hoyt H., Xie J., McMahon G., Bischoping G., Wang D. (2013). Hematite-Based Water Splitting with Low Turn-On Voltages. Angew. Chem. Int. Ed..

[31] Yong K.M.H., Hamann T.W. (2014). Enhanced photocatalytic water oxidation efficiency with Ni(OH)_2_ catalysts deposited on α-Fe_2_O_3_ via ALD. Chem. Commun..

[32] Kim J.Y., Youn D.H., Kang K., Lee J.S. (2016). Highly conformal deposition of an ultrathin FeOOH layer on a hematite nanostructure for efficient solar water splitting. Angew. Chem. Int. Ed..

[33] Yu Q., Meng X., Wang T., Li P., Ye J. (2015). Hematite films decorated with nanostructured ferric oxyhydroxide as photoanodes for efficient and stable photoelectrochemical water splitting. Adv. Funct. Mater..

[34] Yuan Y., Gu J., Ye K.H., Chai Z., Yu X., Chen X., Zhao C., Zhang Y., Mai W. (2016). Combining bulk/surface engineering of hematite to synergistically improve its photoelectrochemical water splitting performance. ACS Appl. Mater. Interfaces.

[35] Chen P.L., Zhong S.M., Cheng X.X., Wang Z.Q., Wang X.T., Fang B.Z. (2024). Steel slag source-derived FeOOH for enhanced BiVO_4_ photoelectrochemical water splitting. J. Colloid Interf. Sci..

[36] Li Y., Mei Q., Liu Z.J., Hu X.S., Zhou Z.H., Huang J.W., Bai B., Liu H., Ding F., Wang Q.Z. (2022). Fluorine-doped iron oxyhydroxide cocatalyst: Promotion on the WO3 photoanode conducted photoelectrochemical water splitting. Appl. Catal. B-Environ..

[37] Fan M.M., Tao Z.Y., Zhao Q., Li J.P., Liu G., Zhao C. (2023). Molecular copper phthalocyanine and FeOOH modified BiVO_4_ photoanodes for enhanced photoelectrochemical water oxidation. Adv. Mater. Technol..

[38] Chen M.H., Chang X.B., Li C., Wang H.Q., Jia L.C. (2023). Ni-Doped BiVO_4_ photoanode for efficient photoelectrochemical water splitting. J. Colloid Interf. Sci..

[39] Li R., Wang Y.M., Chen B.J., Zhang H.M., Yan C., Xu X.F., Humayun M., Debecker D.P., Wang C.D. (2024). Core-shell structured Co(OH)F@FeOOH enables highly efficient overall water splitting in alkaline electrolyte. Int. J. Hydrog. Energy.

[40] Zhu C., Li C., Zheng M., Delaunay J.J. (2015). Plasma-induced oxygen vacancies in ultrathin hematite nanoflakes promoting photoelectrochemical water oxidation. ACS Appl. Mater. Interfaces.

[41] De Faria D.L.A., Silva S.V., De Oliveira M.T. (1997). Raman microspectroscopy of some iron oxides and oxyhydroxides. J. Raman Spectrosc..

[42] Zhu C., Li C., Miao X., Zhao L., Wang Z.Y., Delaunay J.J. (2020). Photoelectrochemical water oxidation performance promoted by a cupric oxide-hematite heterojunction photoanode. Int. J. Hydrog. Energy.

[43] Miao C., Ji S., Xu G., Liu G., Zhang L.D., Ye C.H. (2012). Micro-nano-structured Fe_2_O_3_: Ti/ZnFe_2_O_4_ heterojunction films for water oxidation. ACS Appl. Mater. Interfaces.

[44] Yamashita T., Hayes P. (2008). Analysis of XPS spectra of Fe^2+^ and Fe^3+^ ions in oxide materials. Appl. Surf. Sci..

[45] Li Z., Wu J., Liao L., He X., Huang B., Zhang S., Wei Y., Wang S., Zhou W. (2022). Surface engineering of hematite nanorods photoanode towards optimized photoelectrochemical water splitting. J. Colloid Interf. Sci..

[46] Yang P., Ding Y., Lin Z., Chen Z.W., Li Y.Z., Qiang P.F., Ebrahimi M., Mai W.J., Wong C.P., Wang Z.L. (2014). Low-cost high-performance solid-state asymmetric supercapacitors based on MnO_2_ nanowires and Fe_2_O_3_ nanotubes. Nano Lett..

[47] Grätzel M. (2001). Photoelectrochemical cells. Nature.

[48] Quang N.D., Van P.C., Majumder S., Jeong J.R., Kim D., Kim C. (2022). Rational construction of S-doped FeOOH onto Fe_2_O_3_ nanorods for enhanced water oxidation. J. Colloid Interf. Sci..

[49] Wang Q., Zong X., Tian L., Han Y., Ding Y., Xu C., Tao R., Fan X. (2022). Fe_2_O_3_/FePO_4_/FeOOH ternary stepped energy band heterojunction photoanode with cascade-driven charge transfer and enhanced photoelectrochemical performance. ChemSusChem.

